# Rad51 Nucleoprotein Filament Disassembly Captured Using Fluorescent *Plasmodium falciparum* SSB as a Reporter for Single-Stranded DNA

**DOI:** 10.1371/journal.pone.0159242

**Published:** 2016-07-14

**Authors:** Eric Parker Davenport, Derek F. Harris, Sofia Origanti, Edwin Antony

**Affiliations:** 1 Department of Chemistry and Biochemistry, Utah State University, Logan, Utah, United States of America; 2 Department of Biological Sciences, Marquette University, Milwaukee, Wisconsin, United States of America; Tulane University Health Sciences Center, UNITED STATES

## Abstract

Single-stranded DNA binding (SSB) proteins coordinate DNA replication, repair, and recombination and are critical for maintaining genomic integrity. SSB binds to single-stranded DNA (ssDNA) rapidly and with very high affinity making it a useful molecular tool to detect free ssDNA in solution. We have labeled SSB from *Plasmodium falciparum* (*Pf*-SSB) with the MDCC (7-diethylamino-3-((((2-maleimidyl)ethyl)amino)-carbonyl)coumarin) fluorophore which yields a four-fold increase in fluorescence upon binding to ssDNA. *Pf*-SSB^MDCC^ binding to DNA is unaffected by NaCl or Mg^2+^ concentration and does not display salt-dependent changes in DNA binding modes or cooperative binding on long DNA substrates. These features are unique to *Pf*-SSB, making it an ideal tool to probe the presence of free ssDNA in any biochemical reaction. Using this *Pf*-SSB^MDCC^ probe as a sensor for free ssDNA, we have investigated the clearing of preformed yeast Rad51 nucleoprotein filaments by the Srs2 helicase during HR. Our studies provide a rate for the disassembly of the Rad51 filament by full length Srs2 on long ssDNA substrates. Mutations in the conserved 2B domain in the homologous bacterial UvrD, Rep and PcrA helicases show an enhancement of DNA unwinding activity, but similar mutations in Srs2 do not affect its DNA unwinding or Rad51 clearing properties. These studies showcase the utility of the *Pf*-SSB probe in mechanistic investigation of enzymes that function in DNA metabolism.

## Introduction

**Fig 1:**
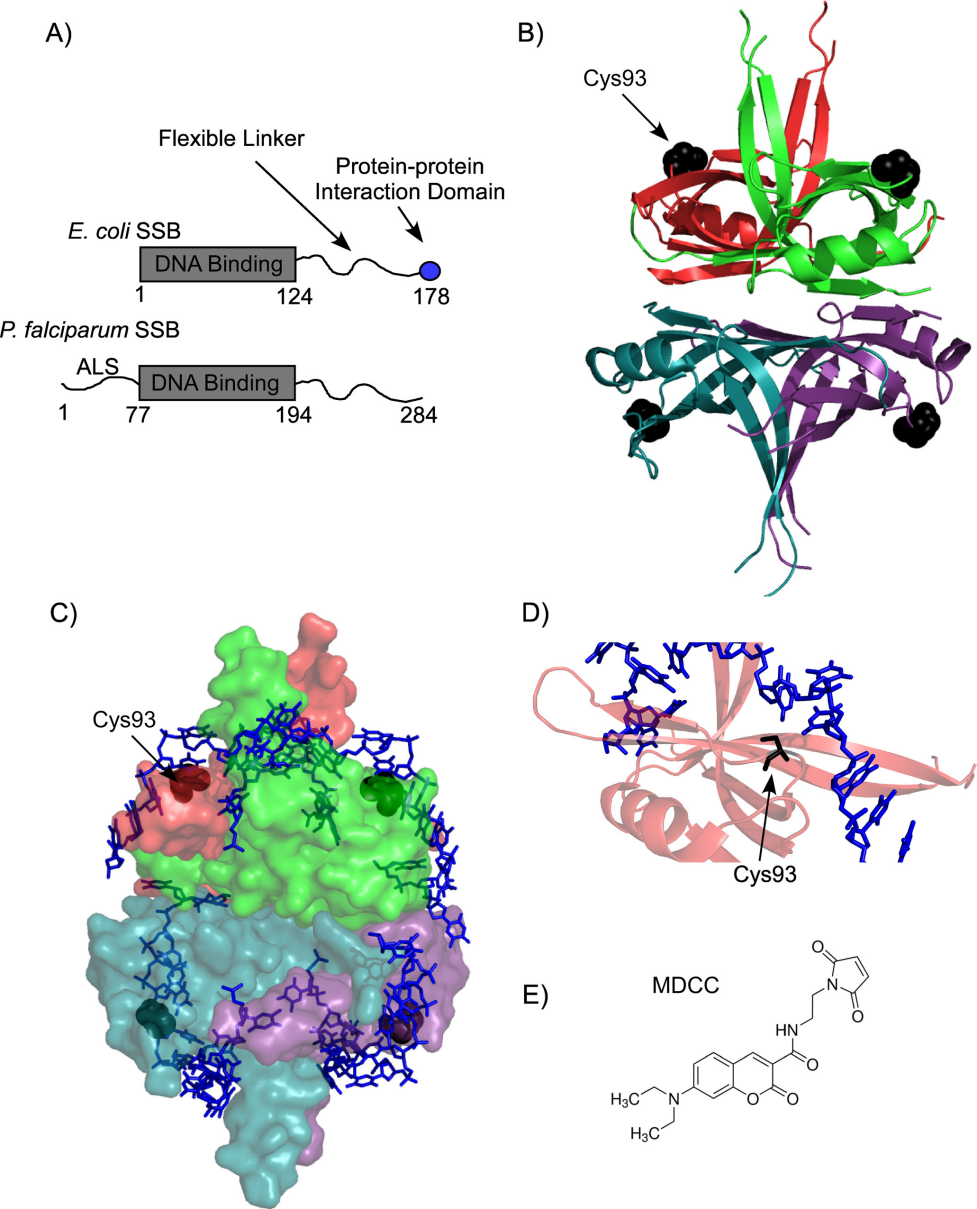
Domain organization of *Pf*-SSB. (**A**) Schematic representation of the DNA binding, protein-protein interaction and linker regions of *E*. *coli* and *P*. *falciparum* SSB. *Pf*-SSB also has an apicoplast localization signal (ALS), which is not required for its DNA binding function. The numbers denote positions of the amino acids at the beginning and end of each domain. (**B**) Individual subunits of the homotetrameric DNA binding domain are depicted as cartoon representation in the crystal structure of *Pf*-SSB. The Cys93 residues used for attachment of the fluorophore are shown as black spheres. (**C**) *Pf*-SSB is shown as surface representation with two (dT)_35_ DNA molecules (blue stick representation) wrapped around the homotetramer. (**D**) The proximity of Cys93 (black stick) to the bound DNA in *Pf*-SSB is highlighted. (**E**) Structure of the MDCC (7-diethylamino-3-((((2-maleimidyl)ethyl)amino)-carbonyl)coumarin) fluorophore used to label *Pf*-SSB. Images of the *Pf*-SSB structure were generated using PDB ID: 3ULP.

Single-stranded DNA binding (SSB) proteins play essential roles in DNA replication, repair, recombination and replication restart, and are found in all domains of life[1]. They bind to single-stranded DNA (ssDNA) with very high selectivity and affinity, and in the cell they protect ssDNA from nucleolytic attacks[2–4]. In addition, they act as a central hub to coordinate various processes essential for maintaining genomic integrity by binding and recruiting a host of DNA replication and repair proteins[5]. The *E*. *coli* SSB (*Ec*-SSB) protein has been shown to mediate protein-protein interactions with up to fourteen different proteins[5]. *Ec*-SSB is the most characterized representative in this class of SSBs and functions as a homotetramer[6]. Each subunit contains a conserved DNA binding domain and a C-terminal 9 amino acid tip that mediates the various protein-protein interactions[7,8]. These two functional domains are connected by a non-conserved intrinsically disordered linker (Fig 1A)[9]. Slight variations to this structural organization are observed in the organelle-based eukaryotic homotetrameric SSB proteins. Mitochondrial SSB (mtSSB) for example contains only the conserved DNA binding domain, whereas SSBs found in the apicoplast of eukaryotic parasites such as *Plasmodium falciparum* and *Toxoplasma gondii*, have the DNA binding core and the disordered linker, but do not contain the protein-protein interaction tip (Fig 1A)[10,11].

Crystal structures of homotetrameric SSB proteins from various organisms show a remarkable similarity in the organization of the DNA binding cores, suggesting a common mechanism of interaction with ssDNA[7,11–15]. *Ec*-SSB binds to ssDNA in multiple DNA binding modes that differ in the number of nucleotides occluded by the tetramer. (SSB)_35_ and (SSB)_65_ are two such modes where the subscript denotes the average number of ssDNA nucleotides engaged by the tetramer[16]. These binding modes are observed when the NaCl or Mg^2+^ concentration is varied in solution. At high salt concentrations (e.g., 200 mM NaCl) the (SSB)_65_ mode is favored where all four subunits in the tetramer bind to DNA. In this mode, *Ec*-SSB has been shown to bind as beads-on-a-string on long ssDNA substrates due to limited cooperativity between the tetramers[17–20]. Interestingly, under these conditions, SSB has been shown to perform numerous activities associated with DNA repair and recombination including, diffusion along ssDNA[21,22], unwinding of hairpins[23] and to promote the formation of RecA nucleoprotein filaments[21]. At lower NaCl concentrations (e.g., 20 mM NaCl), the (SSB)_35_ mode is observed where only two of the four subunits are bound to DNA. SSB tetramers bind with high cooperativity on long DNA substrates, but within each tetramer binding of DNA to two subunits exerts negative cooperativity on the other two subunits[18,20,24]. The complex (SSB)_35_ mode is thought to promote SSB binding and function during DNA replication[25].

SSB from the malarial parasite *Plasmodium falciparum* (*Pf*-SSB) shares a high degree of structural homology with *Ec*-SSB in the homotetrameric DNA binding core[10,11,26]. However, it does not contain the conserved protein-protein interaction tip. The intrinsically disordered linker in *Ec*-SSB tends to adopt more globular conformations, whereas in *Pf*-SSB they tend to be more extended random coils in structure[9]. The crystal structure of *Pf*-SSB shows a remarkable similarity to the *Ec*-SSB structure with ssDNA wrapped around the DNA binding core (Fig 1C), which occludes ~55–62 nt of ssDNA[10,11]. *Pf*-SSB binds to ssDNA with very high affinity (K_a_ >10^10^ M^-1^)[10]. More striking are the differences in the DNA binding properties between the two proteins with respect to salt concentrations in the reaction: *Pf*-SSB displays no evidence of salt-dependent cooperative binding on long DNA molecules[10]. Irrespective of the NaCl concentration in the reaction, *Pf*-SSB binds to two (dT)_35_ or one (dT)_70_ molecule with similar affinity[10,11]. This feature is unique to *Pf*-SSB and is not found in any other bacterial SSBs tested to date. In addition, similar to *Ec*-SSB, *Pf*-SSB can be purified with relative ease and is stable in a wide assortment of buffers. While biochemical explorations of *Pf*-SSB have shed insight on its DNA binding properties, its precise function in the apicoplast is unknown. A role in the replication of apicoplast DNA has been inferred[26]. Whether *Pf*-SSB coordinates DNA repair and recombination in the apicoplast remains to be ascertained; but is not the subject of investigation in this report.

Fluorescently labeled versions of *Ec*-SSB and *Bacillus subtilis* SSB have been developed as real-time probes for ssDNA in biochemical assays[22,27,28]. However, one has to take into account the buffer-dependent variations and cooperative behavior of these SSBs to adequately interpret results. The unique NaCl concentration-resistant DNA binding properties of *Pf*-SSB along with the loss of all the positive and negative cooperative binding features make it an attractive target for ssDNA probe development.

Here, we describe the development of a MDCC-labeled *Pf*-SSB protein (*Pf*-SSB^MDCC^) as a probe for ssDNA. Using this probe, we have captured the interplay between Srs2 and Rad51 in homologous recombination (HR). Rad51 is the central engine for HR and functions by forming a nucleoprotein filament on the resected ssDNA[29,30]. Srs2 is a superfamily-1 (SF1) helicase that functions as an anti-recombinase by disassembling Rad51 molecules from DNA, a process termed *filament clearing*[31–33]. During filament clearing, Srs2 physically interacts with Rad51, stimulating ATP hydrolysis within Rad51 and causing it to dissociate from the DNA [33,34]. Earlier studies of filament clearing by Srs2 were carried out on short DNA oligonucleotide substrates using fluorescently end-labeled DNA substrates[33,35]. When Rad51 binds to these DNA substrates, a change in fluorescence occurs corresponding to filament formation. Disappearance of this signal in the presence of Srs2 was corroborated with filament disassembly. An inherent limitation to these assays is the length of the DNA (< 120 nt in length). HR often occurs on DNA ~1–2 kb in length and the experimental ability to measure nucleoprotein filament dynamics on long DNA substrates would be immensely helpful to study events in HR[36,37]. Using *Pf*-SSB^MDCC^ as a reporter for free ssDNA in the reaction, here we report an assay to monitor the disassembly of Rad51 by Srs2 on long (6400 nt in length), circular m13ssDNA substrates. Using this assay, we report the filament clearing activity of full length Srs2 and a Srs2^DK-AA^ variant carrying mutations in its 2B domain. Our studies reveal that the 2B domain in Srs2 serves functions that appear distinct than in UvrD, PcrA and Rep, the prokaryotic homologs of Srs2.

## Results

### Generation of the fluorescent *Pf*-SSB^MDCC^ probe

**Fig 2:**
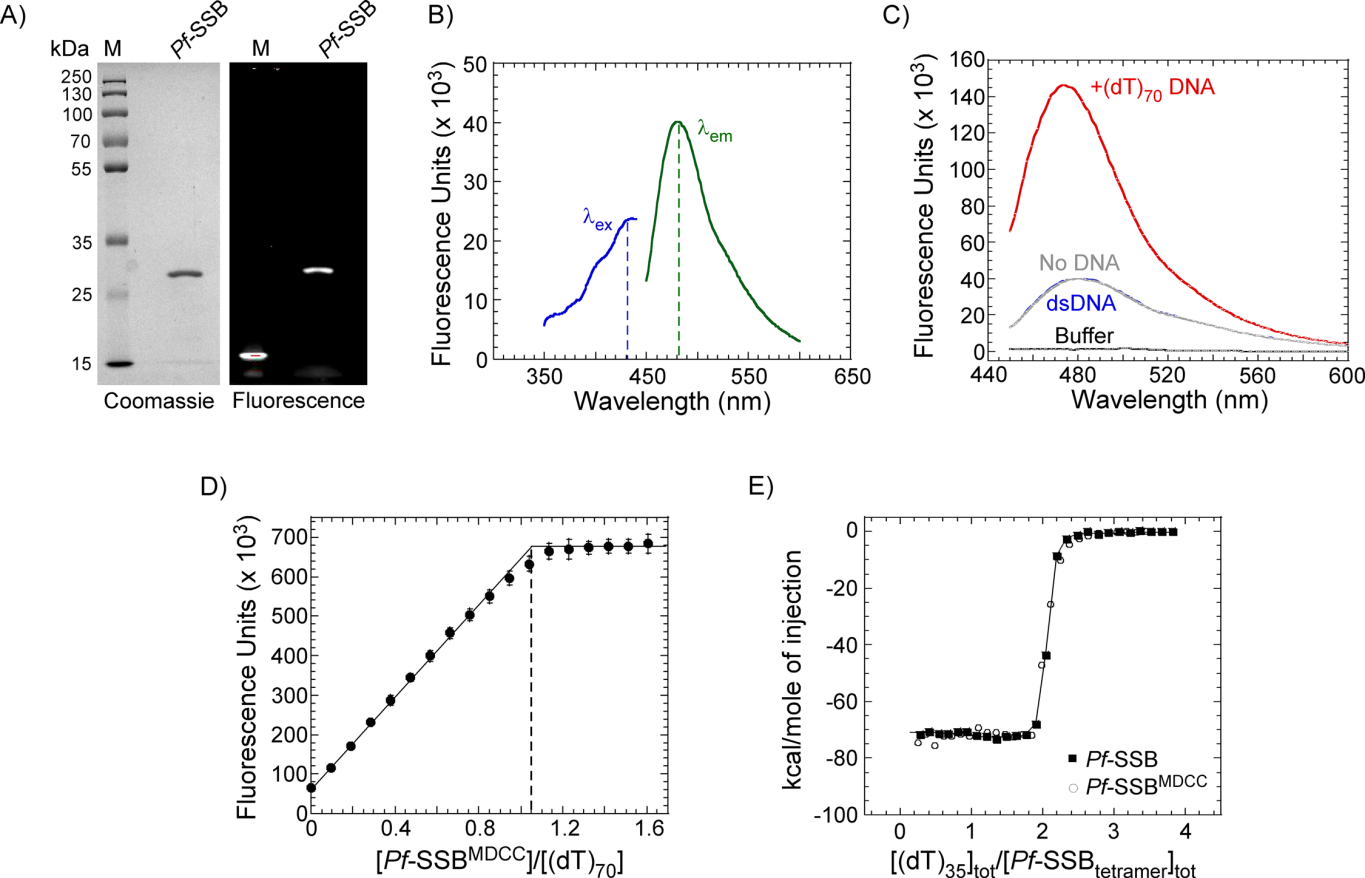
DNA binding properties of *Pf*-SSB^MDCC^. (**A**) Purified *Pf*-SSB^MDCC^ was analyzed on a 12% SDS-PAGE gel and imaged after staining with coommassie dye or detected using fluorescence imaging. M denotes the protein ladder. (**B**) Excitation (blue, λ_ex_) and emission (green, λ_em_) spectra of 1 μM *Pf*-SSB^MDCC^ are shown. The dotted lines correspond to an excitation and emission maxima of 430 nm and 482 nm, respectively. (**C**) 1 μM *Pf*-SSB^MDCC^ was excited at 430 nm and emission spectra were measured in the absence of DNA (grey) and in the presence of a 125 bp dsDNA (blue) or an oligo-dT 70 nt ssDNA (red). A four-fold increase in *Pf*-SSB^MDCC^ fluorescence is observed in the presence of the (dT)_70_ ssDNA oligonucleotide. (**D**) Fluorescence titration of *Pf*-SSB^MDCC^ with increasing concentrations of ssDNA [(dT)_70_]. *Pf*-SSB^MDCC^ binds stoichiometrically, with one SSB tetramer binding to one (dT)_70_ oligonucleotide as denoted by the dotted line. The mean values and standard errors from three independent experiments are shown. (**E**) Isothermal calorimetric measurement of changes in enthalpy associated with binding of two (dT)_35_ molecules to *Pf*-SSB and *Pf*-SSB^MDCC^ are shown. Both proteins bind stoichiometrically with similar observed heat changes ΔH_*obs*_ = -73.1±0.2 kcal mol^-1^ and -71.8±0.2 kcal mol^-1^ for *Pf*-SSB and *Pf*-SSB^MDCC^, respectively. The mean values and standard errors from three independent experiments are shown.

*Pf*-SSB contains an N-terminal apicoplast localization signal (ALS), a DNA binding core and an unstructured C-terminal region (Fig 1A)[26]. The ALS is not required for DNA binding and also prevents proper expression in *E*. *coli* overexpression systems, and hence is not part of the protein investigated here[11]. The DNA binding core is structurally conserved in the homotetrameric class of SSBs and in the case of *Pf*-SSB contains a single Cys residue at position 93 in each subunit (Fig 1B). Cys93 is surface exposed in the crystal structure and is positioned adjacent to the bound DNA (Fig 1C and 1D)[11]. Purified *Pf*-SSB was labeled with MDCC (7-diethylamino-3-((((2-maleimidyl)ethyl)amino)-carbonyl)coumarin) fluorophore (Fig 1E) with >95% labeling efficiency (Fig 2A). MDCC-labeled *Pf*-SSB protein (*Pf*-SSB^MDCC^) shows a fluorescence profile with an excitation and emission maxima of 430 nm and 482 nm, respectively (Fig 2B). The same labeling efficiency was also observed when *Pf*-SSB was labeled with Alexa-555 (not shown). These results suggest that Cys93 is solvent exposed and accessible to complete labeling with maleimide chemistry. MDCC is a relatively small dye and has been shown to be a sensitive fluorophore when tethered onto protein-based probes to detect the binding of ADP[38,39], inorganic phosphate[40,41] and nucleotides[42]. While data obtained with MDCC-labeled *Pf*-SSB are reported here, similar data are observed when *Pf*-SSB is labeled with Alexa-555 (data not shown).

### *Pf*-SSB^MDCC^ binds to DNA with very high affinity and is not influenced by NaCl concentration

*Pf*-SSB has an occluded site size of ~55–62 nt and a (dT)_70_ oligonucleotide completely wraps around the homotetrameric DNA binding core[10,11]. *Pf*-SSB^MDCC^ binds to ((dT)_70_) which results in a ~4-fold increase in fluorescence of the attached fluorophore (Fig 2C). Control experiments show that the binding of *Pf*-SSB^MDCC^ is specific to ssDNA. Addition of double-stranded DNA (dsDNA) to *Pf*-SSB^MDCC^ does not elicit a change in fluorescence (Fig 2C). Unlabeled *Pf*-SSB binds to ssDNA stoichiometrically to a (dT)_70_ oligonucleotide with high affinity (K_obs_ >10^10^ M^-1^)[10]. *Pf*-SSB^MDCC^ binds to a (dT)_70_ substrate stoichiometrically with each tetramer binding to one (dT)_70_ molecule (Fig 2D**)**. In addition, ITC measurements of ssDNA binding to both the unlabeled and labeled *Pf*-SSB yield similar changes in heat capacity, ΔH_*obs*_ = -73.1±0.2 kcal mol^-1^ and -71.8±0.2 kcal mol^-1^ for *Pf*-SSB and *Pf*-SSB^MDCC^, respectively. These data are consistent with DNA binding parameters previously reported for *Pf*-SSB[10] and suggest that the fluorophore does not interfere with the DNA binding properties of *Pf*-SSB, and can be used as a reliable reporter of free ssDNA in a reaction.

**Fig 3:**
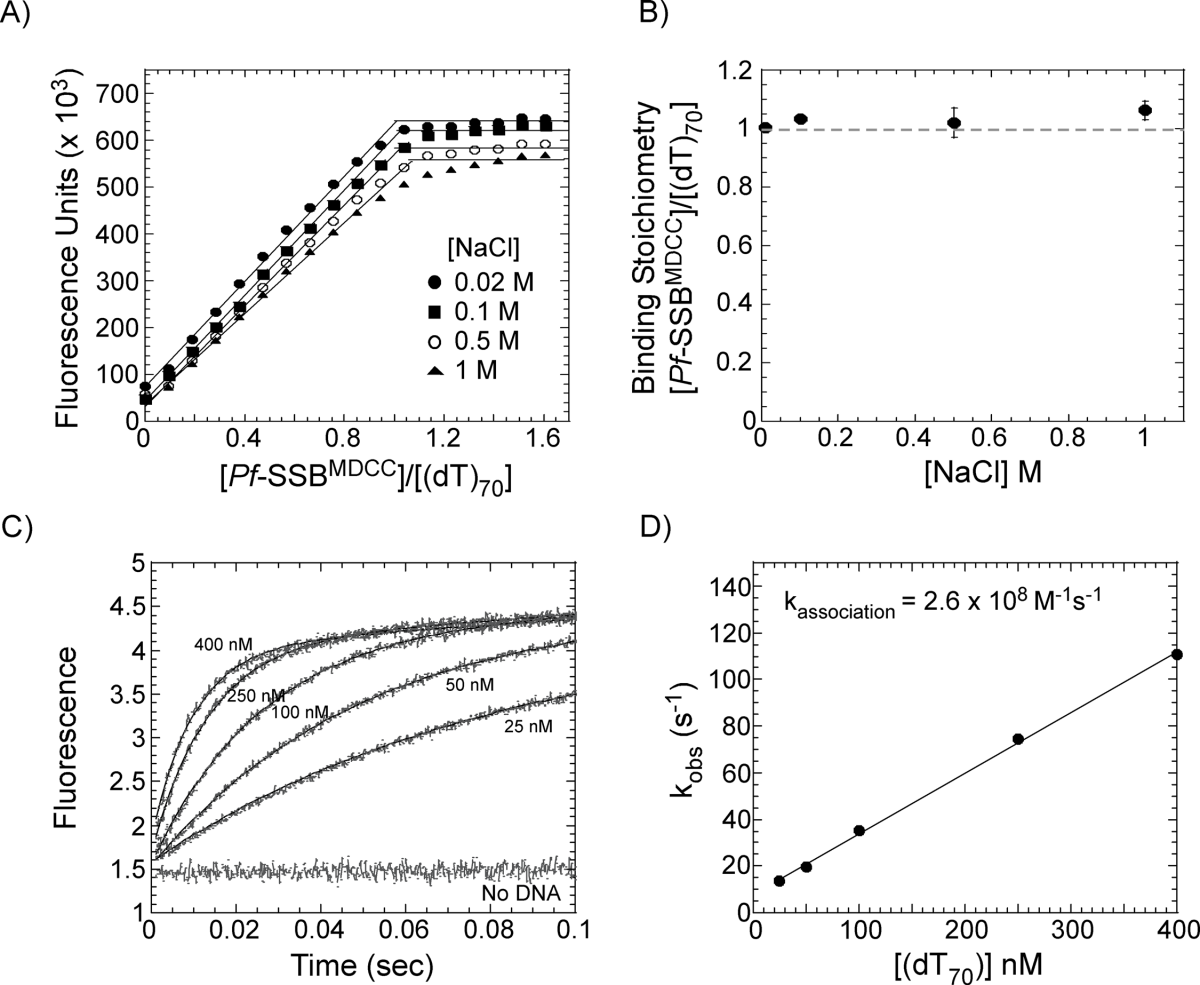
*Pf*-SSB^MDCC^ binds stoichiometrically to ssDNA over a wide range of NaCl concentrations. (**A**) Fluorescence titration of *Pf*-SSB^MDCC^ with increasing concentrations of ssDNA [(dT)_70_] in the presence of increasing concentrations of NaCl. Experiments were performed in 20 mM Tris-Cl, pH 8, 0.1 mM EDTA, and 1 mM TCEP with either 0.02 (●), 0.1 (■), 0.5 (○) or 1M (▲) NaCl in the reaction. (**B**) Stoichiometry of [*Pf*-SSB bound]/[(dT)_70_] under various NaCl conditions from A is plotted as a function of [NaCl] and shows no significant change in binding stoichiometry over a wide range of NaCl concentrations. The mean values and standard errors from three independent experiments are shown. (**C**) Stopped-flow analysis of *Pf*-SSB^MDCC^ binding to (dT)_70_ ssDNA. Rapid binding of *Pf*-SSB^MDCC^ to ssDNA is observed as increasing concentrations of (dT)_70_ are mixed with a fixed concentration of *Pf*-SSB^MDCC^ (20 nM). Data were fit to a single exponential equation and (**D**) the k_obs_ (s^-1^) from the fits were plotted as a function of DNA concentration yielding an apparent association rate constant 2.6 x 10^8^ M^-1^s^-1^.

Since *Pf*-SSB is relatively refractory to changes in NaCl concentration in the reaction, we next tested if the binding of *Pf*-SSB^MDCC^ to ssDNA is affected by [NaCl]. Binding of *Pf*-SSB^MDCC^ to a (dT)_70_ ssDNA substrate was measured at 0.02, 0.1, 0.5 or 1 M NaCl in the reaction. In each of these experiments, one *Pf*-SSB^MDCC^ molecule stoichiometrically binds to one (dT)_70_ ssDNA molecule (Fig 3A and 3B). These results show that the fluorescently labeled *Pf*-SSB^MDCC^ protein is also refractory to changes in [NaCl]. A small decrease in total fluorescence is observed as the [NaCl] is increased in the reaction, suggesting that the quantum yield of the fluorophore is slightly sensitive to buffer conditions, but the ssDNA binding properties are relatively unaffected (Fig 3A). Similar results were observed when the [MgCl_2_] concentration was varied from 0–10 mM in the reaction (not shown). These results showcase the versatility of *Pf*-SSB^MDCC^ as a probe for ssDNA, and in this respect, is better suited for probe development than *E*. *coli* SSB and SSB proteins from other bacterial species tested thus far.

### *Pf*-SSB^MDCC^ binds rapidly to ssDNA

A probe for ssDNA should possess rapid binding capability. *Pf*-SSB has been shown to possess fast ssDNA binding kinetics. To test whether the attachment of the fluorophore affects the DNA binding kinetics, we mixed increasing concentrations of ssDNA with *Pf*-SSB^MDCC^ in a stopped-flow instrument and monitored the change in fluorescence (Fig 3C). *Pf*-SSB^MDCC^ rapidly binds to DNA as observed by the saturation of the fluorescence signal well before 100 msec. The data are fit to a single exponential function and analysis of the k_*obs*_ as a function of [(dT)_70_] yields an apparent association rate constant of 2.6 x 10^8^ M^-1^s^-1^ (Fig 3D). These results suggest that *Pf*-SSB^MDCC^ binding to ssDNA is diffusion limited and is ideal as a probe for free ssDNA in any reaction.

### Preformed Rad51 filaments are unaffected by *Pf*-SSB^MDCC^

**Fig 4:**
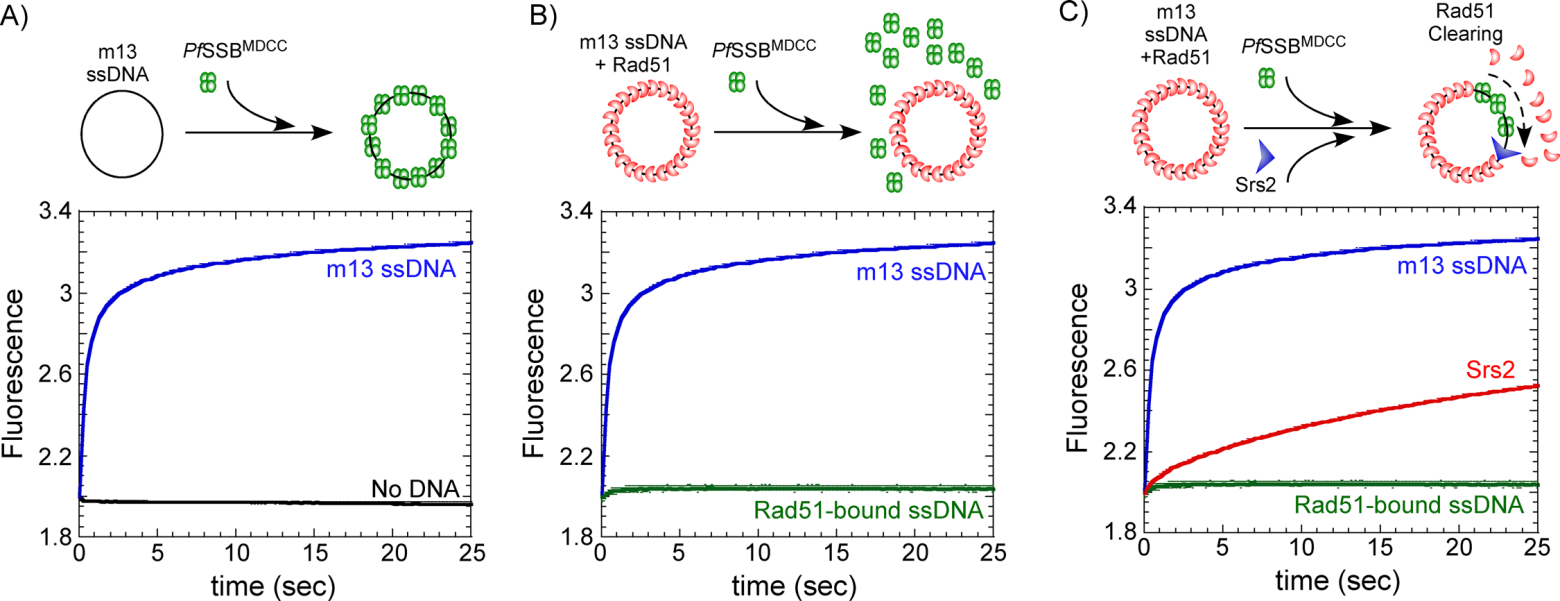
Rad51 filament clearing by Srs2 captured by *Pf*-SSB^MDCC^. (**A**) Stopped-flow measurement of *Pf*-SSB^MDCC^ (80 nM) binding to m13 circular ssDNA (3 μM nucleotides) is shown by a rapid increase in fluorescence (blue trace). No change in fluorescence is observed in the absence of ssDNA (black trace). (**B**) When m13ssDNA (3 μM nucleotides) is pre-coated with Rad51 (3 μM)in the presence of ATP (3 mM) and then mixed with *Pf*-SSB^MDCC^ (80 nM), no significant change in fluorescence is observed (green trace) suggesting that *Pf*-SSB^MDCC^ does not gain access to ssDNA when it is completely bound by Rad51 in the form of a nucleoprotein filament. (**C**) Challenging the Rad51 nucleoprotein filament on m13ssDNA with *Pf*-SSB^MDCC^ in the presence of full length Srs2 (25 nM) results in a gradual increase in the fluorescence signal (red). Srs2 clears the Rad51 from the ssDNA yielding free ssDNA for the rapid and tight binding of *Pf*-SSB^MDCC^. Models for the reaction mixing schemes are presented above each data panel.

Rad51 binds to ssDNA and forms the nucleoprotein filament during HR[33,37,43]. Mediator proteins, such as the Srs2 helicase, disassemble Rad51 molecules during HR thereby creating transient gaps in ssDNA[32,33]. In an *in vitro* reaction looking at Rad51 binding and dissociation, these open ssDNA intermediates provide a binding opportunity for a secondary reporter such as *Pf*-SSB^MDCC^. The change in fluorescence upon *Pf*-SSB^MDCC^ binding to ssDNA can be utilized as a measure of Rad51 DNA binding/dissociation kinetics. We have previously reported an apparent rate for filament clearing by a truncated version of Srs2 (lacking a portion of the C-terminal tail—Srs2^1-898^) on short ssDNA oligonucleotides (<125 nt)[33]. Since HR often occurs on DNA ~1 to 2 kb in length, the experimental ability to measure nucleoprotein filament dynamics on longer DNA substrates would be immensely helpful[36,37]. SSB can bind and completely sequester long DNA substrates. To establish the utility of *Pf*-SSB^MDCC^ as a probe for long DNA substrates, we tested the ability of *Pf*-SSB^MDCC^ to bind a 6.4 kb circular m13ssDNA substrate. *Pf*-SSB^MDCC^ binds rapidly to free circular m13ssDNA and displays a robust change in the fluorescence signal (Fig 4A).

To investigate Rad51 filament dynamics on long ssDNA substrates using the *Pf*-SSB^MDCC^, we first preformed the Rad51 filament on m13ssDNA. Yeast Rad51 forms a stable nucleoprotein filament in the presence of ATP. This feature is unique to *S*. *cerevisiae* Rad51 as both human Rad51 and the bacterial homolog RecA have been shown to be in equilibrium between the bound and unbound states in the presence of ATP[44–48]. We used 80 nM *Pf*-SSB^MDCC^ (tetramer concentration) to saturate all the potential ssDNA binding sites when 3 μM total nucleotides of the m13ssDNA were used in the reaction. We used a binding site size of ~60 nt bound/SSB tetramer as a rubric for the reaction (50 nM total SSB binding sites), and complete saturation of the *Pf*-SSB^MDCC^ fluorescence is observed under these conditions (Fig 4A).

In order to be a usable reporter of Rad51 dynamics, *Pf*-SSB^MDCC^ should not interfere with preformed Rad51 filaments. To test this, *Pf*-SSB^MDCC^ was mixed with preformed Rad51 filaments (Rad51 + ATP) in a stopped-flow, and the change in fluorescence was monitored (Fig 4B). 2 to 3 μM Rad51 was sufficient to occupy all the ssDNA in the reaction and we used a site size of ~3 nt/Rad51 (1 μM total Rad51 binding sites) to formulate the conditions for filament formation[49–51]. No change in fluorescence is observed suggesting that *Pf*-SSB^MDCC^ did not displace Rad51and no free ssDNA is available in this scenario for *Pf*-SSB^MDCC^ to bind in the reaction (Fig 4B). In the control experiments without Rad51, *Pf*-SSB^MDCC^ rapidly binds to free ssDNA and an exponential rise in fluorescence signal is observed (Fig 4A and 4B).

### Srs2 rapidly clears Rad51 molecules off ssDNA

Using *Pf*-SSB^MDCC^ and Rad51 nucleoprotein filaments formed on circular m13ssDNA, we tested whether full length Srs2 will clear long nucleoproteins filaments. Preformed Rad51 nucleoproteins were first formed as described above by incubating Rad51, m13ssDNA, ATP and an ATP regenerating system as described in the methods section. These filaments were mixed with Srs2 in a stopped-flow and the change in fluorescence was monitored. A gradual increase in fluorescence is observed in the presence of Srs2 (Fig 4C). Srs2 disassembles Rad51 molecules creating gaps in the ssDNA, which are bound by the *Pf*-SSB^MDCC^ molecules. Srs2 could initiate Rad51 disassembly from multiple positions on the filament and hence a complete model to fit the data cannot be accurately generated. These results show that the *Pf*-SSB^MDCC^ probe can be used as a reporter for measurement of Rad51 clearing during HR. This assay will be useful to measure the effect of other mediator proteins such as the SHU complex on Rad51 filament stability and to investigate the interplay between Srs2 and other mediator proteins on long nucleoprotein filaments[30,43].

### Mutations in the 2B domain of Srs2 do not enhance its DNA unwinding or filament clearing activities

Srs2 is a SF1 helicase capable of unwinding dsDNA[52]. Srs2 is homologous to the bacterial UvrD, Rep and PcrA helicases[53]. The 2B domain in UvrD, Rep and PcrA have been shown to be inhibitory for the DNA unwinding activity of these enzymes[54–60]. Mutations or deletions in the 2B domain stimulate their DNA unwinding activity[58,60]. The role of the 2B domain of Srs2 in DNA unwinding or nucleoprotein filament clearing have not been explored.

**Fig 5:**
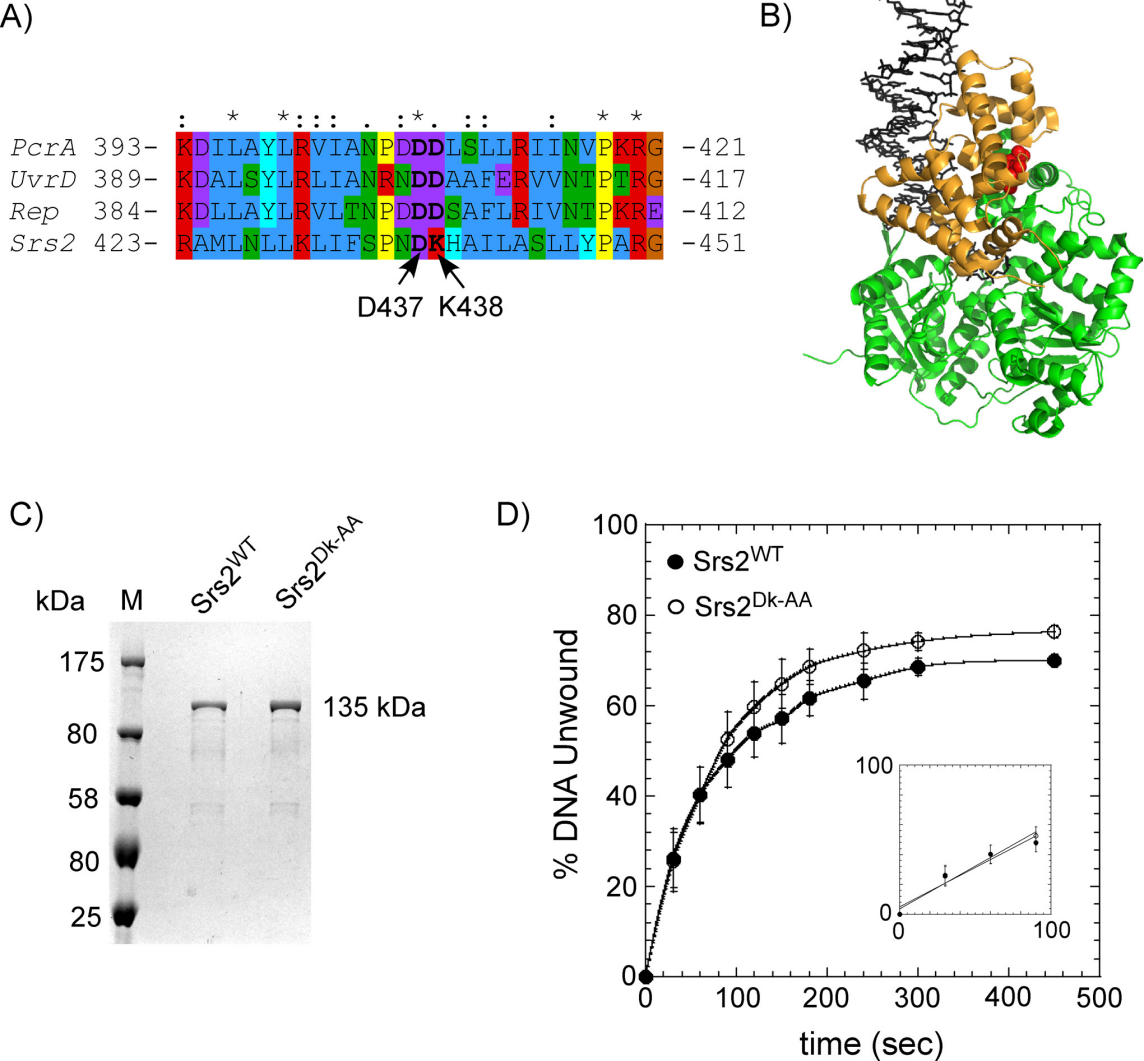
2B domain mutations in Srs2 have no effect on its DNA unwinding capabilities. (**A**) Alignment of the region in the 2B domains from UvrD, Rep, PcrA and Srs2. D437 and K438 in Srs2 align with D403 and D404 in UvrD, which are mutated in the *uvrD303* phenotype, a hyperactive helicase mutant of UvrD. Amino acids are colored according to their physicochemical properties. (**B**) Crystal structure of the UvrD (PDB ID:2IS4; the bacterial Srs2 homolog) is shown with the 2B domain colored gold. The 2B domain is in the ‘closed conformation’ when bound to the unwinding DNA substrate. The DNA is shown as sticks (black) and the D403-D404 residues are shown as red spheres. (**C**) SDS-PAGE analysis of the purified full length Srs2^WT^ and Srs2^DK-AA^ proteins. (**D**) Unwinding kinetics of a DNA substrate (25bp dsDNA with a 16 nt 3' ssDNA overhang) by Srs2^WT^ and Srs2^DK-AA^. No discernable difference in unwinding kinetics is observable between the two proteins. Fitting the linear portion of the data (insert) yield unwinding rates of 0.026 s^-1^ and 0.028 s^-1^ for the Srs2^WT^ and Srs2^DK-AA^ proteins, respectively. The mean values and standard errors from three independent experiments are shown.

In *uvrd303*, a hyperactive helicase variant, two aspartate residues (D403, D404) are mutated to alanine (Fig 5A)[61–63]. They reside in the 2B domain and are thought to participate in controlling the transition between the ‘closed’ and ‘open’ conformations of this domain (Fig 5B)[61]. The two aspartates are conserved in UvrD, Rep and PcrA, but only the first aspartate residue is conserved in Srs2 (D437). The second aspartate is replaced with a Lysine in *S*. *cerevisiae* Srs2 (Fig 5A). To test the significance of these differences in this region, we generated a Srs2 variant by substituting D437 and K438 with two alanine residues and purified the Srs2^DK-AA^ variant (Fig 5C). We next tested the difference in DNA unwinding rates of the full length Srs2^WT^ and Srs2^DK-AA^ proteins on a DNA substrate with a 25bp dsDNA with a 16 nt 3' ssDNA overhang, as previously described[35]. Both proteins unwind DNA with similar kinetics (Fig 5D), unwinding the DNA substrate at ~0.025 s^-1^. These results suggest that these residues in the 2B domain of Srs2 do not play a role in DNA unwinding compared to the reported observations of enhanced DNA unwinding for the bacterial homologs UvrD, PcrA and Rep[54,57,58]. Other fluorescent SSB probes have been used as a tool to measure DNA unwinding kinetics of helicases[27], and *Pf*-SSB^MDCC^ can also be used to measure the DNA unwinding kinetics of Srs2 (Davenport *et*. *al*., unpublished data).

**Fig 6:**
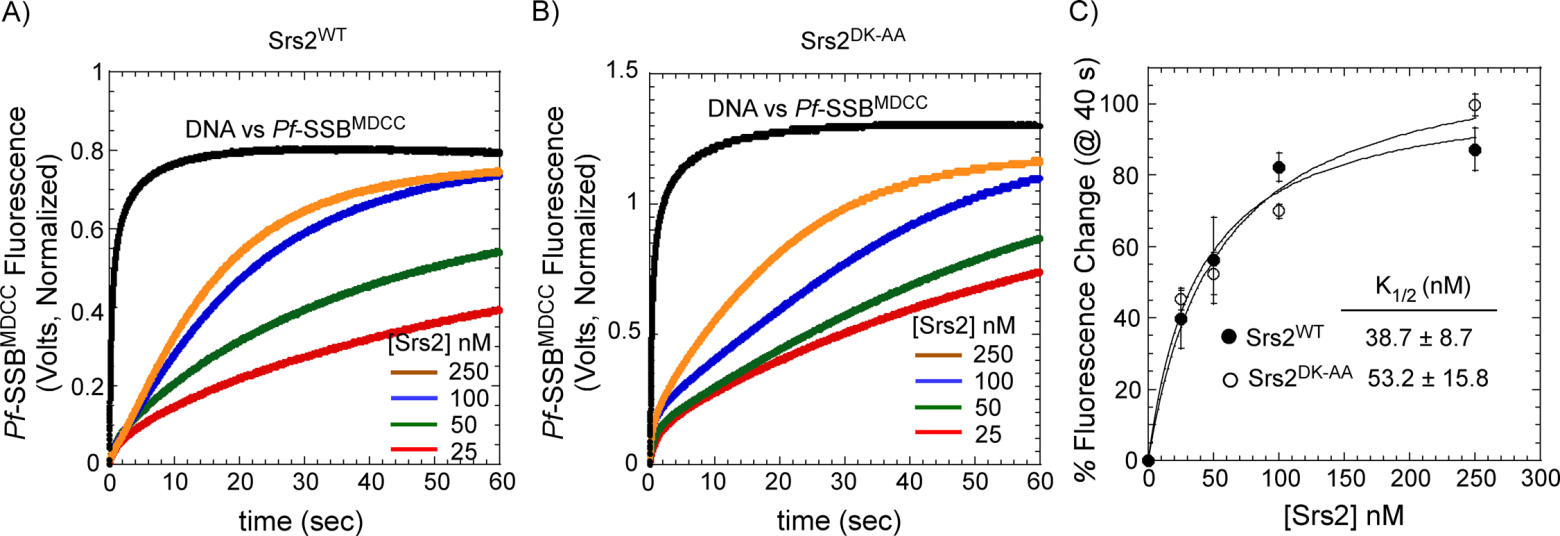
Nucleoprotein filament clearing activity of Srs2 is unaffected by mutations in its 2B domain. Stopped-flow analysis of Rad51 nucleoprotein filament clearing with increasing concentrations of (**A**) Srs2^WT^ and (**B**) Srs2^DK-AA^ were measured using *Pf*-SSB^MDCC^ as a reporter for free ssDNA in the reaction. (**C**) The percent change in *Pf*-SSB^MDCC^ fluorescence at time = 40 sec from A and B are plotted against Srs2 concentration and shows a hyperbolic relationship. The K_1/2_ for Rad51 filament clearing was obtained by fitting the data to a hyperbola and shows that both proteins clear Rad51 filaments with similar efficiency. The mean values and standard errors from three independent experiments are shown.

We next tested whether these residues in the 2B domain play a role in the filament clearing activity of Srs2. Using *Pf*-SSB^MDCC^ as a probe to monitor Rad51 nucleoprotein filament disassembly, we measured the kinetics of filament clearing of Srs2^WT^ and Srs2^DK-AA^. Filament clearing was measured by challenging preformed nucleoprotein filaments on circular m13ssDNA with increasing concentrations of Srs2 and *Pf*-SSB^MDCC^, and the change in fluorescence was measured in a stop flow instrument. To measure the Rad51 filament clearing activity, the total fluorescence from a specific time point in the reaction (e.g., at 40 sec; Fig 6) was plotted as a function of [Srs2] and an apparent K_1/2_ for clearing was calculated by fitting the data to a Menton hyperbola (Fig 6C). Srs2^WT^ and Srs2^DK-AA^ both cleared long Rad51 nucleoprotein filaments with relatively similar efficiency: K_1/2_ = 38.7±8.7 and 53.2±15.8 for Srs2^WT^ and Srs2^DK-AA^ respectively. These results suggest that these residues in the 2B domain of Srs2 do not play a key role in the Rad51 clearing mechanism.

## Discussion

The ability to monitor activity of DNA binding proteins on long DNA substrates is useful in mimicking reaction substrates that are encountered in the cell. *Pf*-SSB^MDCC^ is well suited as a tool to report the presence of free ssDNA of varying lengths in a reaction irrespective of the buffer conditions used. The binding of *Pf*-SSB^MDCC^ to DNA is not affected by [NaCl] or [Mg^2+^] in the reaction. In addition, the salt dependent variations in intra-subunit and inter-subunit cooperativity between the tetramers observed for *Ec*-SSB[16,19] are not applicable to *Pf*-SSB[10,11]. Hence, *Pf*-SSB serves as a more versatile probe for ssDNA compared to SSB proteins from *E*. *coli* or SSB proteins from other bacterial origins. *Pf*-SSB^MDCC^ rapidly binds to ssDNA with very high affinity making it an ideal reporter for real-time kinetics of enzyme activity. Here, we have used *Pf*-SSB^MDCC^ to monitor an activity that occurs during homologous recombination.

Formation of the Rad51 filament drives HR and its dissociation hinders HR. Pro-HR mediators promote HR by stabilizing the Rad51 filament whereas anti-HR mediators, such as Srs2, prevent inopportune HR events by removing Rad51 molecules from DNA[43]. Ensemble experiments looking at Rad51 filament disassembly were limited to short DNA substrates end-labeled with fluorophores[33,64]. Recent single molecule experiments using DNA curtains have been able to capture Rad51 dynamics on long DNA substrates[65], but are limited by the need for expensive and custom-built total internal reflection microscopy based instrumentation. *Pf*-SSB^MDCC^ serves as a feasible probe to monitor the disassembly of *S*. *cerevisiae* Rad51 nucleoprotein filaments. We have captured disassembly of Rad51 filaments by full length Srs2 on a 6.4 kb long m13ssDNA circular substrate. Our earlier investigation of Rad51 filament clearing by a truncated version of Srs2 (Srs2^1-898^) yielded a K_1/2_ = 380 nM[33]. Here, we observe a K_1/2_ = 38.7 nM for filament clearing by full length Srs2 on long DNA substrates (Fig 6C**)**. In the earlier study, Rad51 would dissociate and rebind to the free ssDNA and occluded the true efficiency of filament clearing. In the current assay, *Pf*-SSB^MDCC^ serves as a trap for free ssDNA and prevents the rebinding of Rad51, thereby providing a more accurate measure of the filament clearing by Srs2. During HR, the RPA protein (the eukaryotic functional homolog of SSB) serves the role of sequestering free DNA as Rad51 is displaced by Srs2[32,66].

The *Pf*-SSB^MDCC^ based assay for filament clearing was also used to test the role of the Srs2 2B domain. In UvrD and Rep, the 2B domain adopts two specific conformations–an ‘open conformation’ where the 2B domain is situated above the 2A domain, and a ‘closed conformation’ where the 2B domain moves over the 1A domain[54,67]. In the presence of DNA, the 2B domain adopts the closed conformation (Fig 5B)[67–69]. Deletion of the 2B domain stimulates the DNA unwinding activity of both UvrD and Rep suggesting that the 2B domain is inhibitory to unwinding activity[58,60,61]. *uvrD303* is a hyperactive DNA unwinding variant of UvrD carrying two mutations in the 2B domain. D403 and D404 are mutated to alanine residues in this UvrD variant[61] and these residues are highly conserved in the Srs2 homologs (Fig 5A**)**. Since only the first aspartate residue is conserved in Srs2, we generated the Srs2^DK-AA^ variant to investigate the role of the 2B domain. We see no differences in either the DNA unwinding or Rad51 clearing kinetics between Srs2^WT^ and Srs2^DK-AA^ (Fig 6), suggesting that these residues in the 2B domain might be serving a different role in Srs2 and warrants more investigation.

**Fig 7:**
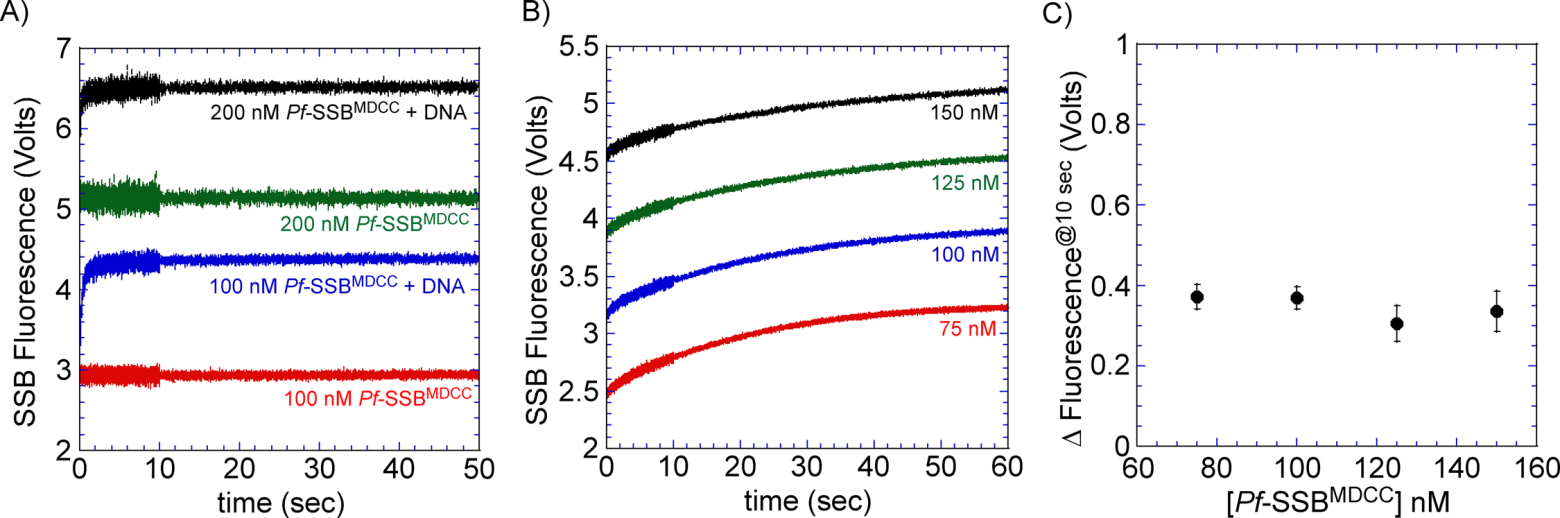
*Pf*-SSB^MDCC^ does not influence the activity of Srs2. (**A**) Change in fluorescence upon mixing varying concentrations of *Pf*-SSB^MDCC^ (100 or 200 nM) with buffer in the presence or absence of m13ssDNA (3 μM nucleotides). (**B**) Rad51 filament clearing by Srs2 was measured in the presence of increasing concentrations of *Pf*-SSB^MDCC^. Preformed Rad51 filaments were rapidly mixed with varying amounts of *Pf*-SSB^MDCC^ (75, 100, 125 or 150 nM) and Srs2 (100 nm) and the change in fluorescence was measured over time. Data were collected over a split time period with 5000 points each assigned to the first 10 sec and remaining 50 sec, respectively. An average of 10 independent traces is shown. (**C**) The normalized change in fluorescence at time = 10 sec was subtracted from time = 0.01 sec and the Δfluorescence^@10sec^ values plotted as a function of [*Pf*-SSB^MDCC^]. No significant change in fluorescence is observed. The mean values and standard errors from three independent experiments are shown.

While SSB proteins are attractive tools as probes for free ssDNA in the reaction, one needs to control for inadvertent effects of SSB on their protein/system of investigation. Performing the experiments with multiple concentrations of probe-SSB and being certain that the measured reaction rates/behavior do not change would address such concerns. An example of such control experiments is presented (Fig 7). Increasing the concentration of the *Pf*-SSB^MDCC^ probe in the reaction results in the enhancement of the total fluorescence signal (Fig 7A). However, the kinetics of filament clearing by Srs2 is independent of probe concentration (Fig 7B and 7C**)** and hence, in this case, is a true reporter of free ssDNA in the reaction.

In summary, *Pf*-SSB^MDCC^ serves as a robust tool to measure the presence of free ssDNA in a reaction. In addition to being a tool to monitor DNA unwinding by DNA helicases, it can be tailored to study events such as the dissociation of Rad51 during homologous recombination. This *Pf*-SSB^MDCC^ probe should be amenable as a tool to monitor the dynamics of other DNA binding enzymes.

## Materials and Methods

### Chemicals and Reagents

All standard laboratory chemicals, phosphocreatine and creatine phosphokinase were purchased from Sigma-Aldrich (St. Louis, MO). MDCC (7-diethylamino-3-((((2-maleimidyl)ethyl)amino)-carbonyl)coumarin) was purchased from Thermo Fisher Scientific (Grand Island, NY).

### DNA

Oligonucleotides used in this study were synthesized by Integrated DNA Technologies (Coralville, IA). Phage m13mp18ssDNA was purchased from New England Biolabs (Ipswich, MA). All ssDNA concentrations were determined spectrophotometrically using the extinction coefficient ε_260_ = 8.1 x 10^3^ M^-1^ (nucleotide) cm^-1^ for oligo(dT) and ε_259_ = 7370 M^-1^ cm^-1^ for m13ssDNA in 20 mM Tris-Cl, pH 8.0.

### Proteins

*Pf*-SSB, *Saccharomyces cerevisiae* Srs2 (full length) and Rad51 proteins were purified as described[10,11,33]. Concentration of *Pf*-SSB was determined using extinction coefficient: ε_280_ = 9.58 ×10^4^ M^-1^ cm^-1^ (*Pf*-SSB tetramer). All SSB concentrations denoted in this study are for the tetramer. Concentration of Srs2 and Rad51 were determined spectroscopically using extinctions coefficients ε_280_ = 82,670 M^-1^cm^-1^ and 12,420 M^-1^cm^-1^ respectively. The Srs2^DK-AA^ mutation was generated using the Q5 site directed mutagenesis kit (NEB, Ipswich, MA) and purified similar to the wild type Srs2 protein.

### Labeling of *Pf*-SSB

750 μl of 400 μM *Pf*-SSB (monomer concentration) was first dialyzed into labeling buffer (50 mM potassium phosphate, pH 7.0, 100 mM NaCl, and 5% v/v glycerol) at 4°C. A 16-fold molar excess of freshly made TCEP was added to the SSB protein prior to labeling. The MDCC fluorophore was resuspended in DMSO and added to the protein at a final molar ratio of 2:1 [MDCC:SSB^monomer^]. The reaction was incubated overnight at 4°C and quenched by adding a 5-fold excess of 2-mercaptoethanol. Excess dye from the reaction was removed by purifying the labeled protein on a Biogel P-4 polyacrylamide gel size exclusion column (Bio Rad Laboratories, Hercules, CA) at 4°C using labeling buffer. Fractions containing labeled SSB were pooled and dialyzed into SSB storage buffer (20 mM Tris-Cl, pH 8.3, 1 mM Na_3_EDTA, 500 mM NaCl, 5 mM 2-mercaptoethanol, and 50%(v/v) glycerol). SSB concentration was measured using ε_280_ = 95,800 M^-1^ cm^-1^ (*Pf*-SSB tetramer) and MDCC concentration was measured using ε_430_ = 49,300 M^-1^ cm^-1^. A correction factor was applied to the final protein concentration to account for the MDCC absorbance at 280 nm: [SSB]-([SSB]*0.3634), yields the correct concentration of *Pf*-SSB^MDCC^. This labeling procedure routinely yielded ~95–99% MDCC-labeled *Pf*-SSB protein. Proteins were stored at -20°C for up to 12 months with no loss in DNA binding activity.

### DNA binding measurements

Binding of *Pf*-SSB to (dT)_70_, was examined in buffer (20 mM Tris-Cl, pH 8, 0.1 mM EDTA, 100 mM NaCl and 1 mM TCEP) by monitoring the change in *Pf*-SSB^MDCC^ fluorescence upon titration with DNA at 25°C (ISS PC1 spectrofluorometer, Champaign, IL) [λ_ex_ = 430 nm (2 nm band-pass), and λ_em_ = 482nm (2–5 nm band-pass)].

### Stopped-flow DNA binding and Rad51 clearing experiments

All the stopped-flow experiments were carried out using an Auto SF-120 Stopped-Flow instrument (Kintek Corp., Snow Shoe, PA) at 25°C or a SX20 Stopped-Flow instrument from Applied Photophysics (Surrey, UK). To monitor the fluorescence changes arising from *Pf*-SSB^MDCC^, samples were excited at 430 nm and emission was monitored using a 450 nm long pass cut-off filter (Newport Inc., Irvine, CA). *Pf*-SSB^MDCC^ binding to ssDNA [(dT)_70_] was measured in buffer (20 mM Tris-Cl, pH 8, 0.1 mM EDTA, 100 mM NaCl and 1 mM TCEP) at 25°C and by rapidly mixing 20 nM *Pf*-SSB^MDCC^ against varying concentrations of (dT)_70_ = 0–400 nM. Data were fit to single exponential equation and the rates were plotted against DNA concentration to obtain an association rate constant. For the filament clearing experiments, Rad51 nucleoprotein filaments were first formed on m13ssDNA by incubating 3 μM Rad51 with m13ssDNA (3 μM nucleotides) in filament buffer (25 mM Tris-Cl, pH 7.7, 1 mM TCEP, 5% v/v glycerol, 10 mM MgCl_2_ an 50 mM NaCl) with 3 mM ATP and an ATP regenerating system (30 mM phosphocreatine and 0.2 mg/ml creatine phosphokinase). These filaments were mixed with reactions containing 80 nM *Pf*-SSB^MDCC^ in the presence or absence of 25–250 nM Srs2. All concentrations noted here are ‘post-mixing’ in the stop flow setup. The fluorescence intensities at a given time point (e.g. 40 sec; Fig 6A and 6B) were normalized against the total fluorescence change for *Pf*-SSB^MDCC^ binding to free m13ssDNA to yield the percent change in fluorescence. These values were then plotted against [Srs2] and the data were fitted to a hyperbola to yield apparent K_1/2_ values for filament clearing. The effect of total [*Pf*-SSB^MDCC^] on filament clearing by Srs2 was measured similarly, but the concentration of SSB was increasing in each experiment as denoted (75, 100, 125 or 150 nM). The change in fluorescence at 10 sec (Δfluorescence^@10sec^) was calculated by subtracting the fluorescence value at t = 10 sec with the value measured at t = 0.01 sec for each trace observed in the dataset.

Isothermal calorimetric measurement of *Pf*-SSB binding to ssDNA was performed using a VP-ITC microcalorimeter (GE Inc., Piscataway, NJ) as described previously[10]. Briefly, 1 μM of *Pf*-SSB *or Pf*-SSB^MDCC^ was incubated in the cell in buffer (20 mM Tris-Cl, pH 8, 0.1 mM EDTA, 200 mM NaCl and 1 mM TCEP) and titrated with (dT)_35_ (24 μM concentrations in the syringe). The reference heats of dilutions were determined by titrating DNA solution into the buffer in the cell. Proteins and DNA were dialyzed extensively into the reaction buffer (4 buffer changes) prior to the experiments. The ITC data for *Pf*-SSB binding to two molecules of (dT)_35_ were analyzed as described[10].

### DNA unwinding assay

The substrate for DNA unwinding was prepared by annealing EA-T: 5′-CGATCGTCC-TCTAGACAGCTTACGC-3′ labeled with γ-^32^P-ATP (Perkin Elmer) and polynucleotide kinase (NEB) and EA-B: 5′-GCGTAAGCTGTCTAGAGGACGATCG[T]_16_ yielding a 25 bp dsDNA with a 16 nt 3′ ssDNA overhang. DNA unwinding by Srs2 was measured by the incubating 10 nM of the DNA with 250 nM Srs2 and monitoring the displacement of the labeled EA-T strand. Reactions were performed in DNA unwinding buffer [20 mM KHPO_4_ (pH 8.0), 120 mM NaCl, 10 mM MgCl_2_, 100 μg/ml bovine serum albumin and 0.2 mM β-mercaptoethanol] at 30°C and initiated by adding ATP (2 mM) to the preformed Srs2-DNA complex. Samples were removed at the denoted time points and quenched with stop buffer [100 mM EDTA (pH 8.0), 20% v/v glycerol and 0.4% SDS]. The dsDNA substrate and dissociated ssDNA product strand were resolved by EMSA in a 10% TBE (*T*ris–*b*orate–*E*DTA) acrylamide gel (100 V at 25°C in 1× TBE), dried and quantified using a phosphor imager.
